# Social determinants of health and cognitive function: A cross‐sectional study among men without dementia

**DOI:** 10.1002/brb3.3235

**Published:** 2023-08-31

**Authors:** Kayla B. Corney, Julie A. Pasco, Amanda L. Stuart, Bianca E. Kavanagh, Mohammadreza Mohebbi, Sophia X. Sui, Lana J. Williams

**Affiliations:** ^1^ Deakin University, IMPACT – Institute for Mental and Physical Health and Clinical Translation, School of Medicine Deakin University Geelong Australia; ^2^ Barwon Health University Hospital Geelong Geelong Australia; ^3^ Department of Medicine‐Western Health The University of Melbourne St Albans Australia; ^4^ Faculty of Health, Biostatistics Unit Deakin University Geelong Australia; ^5^ Deakin Rural Health, School of Medicine Deakin University Warrnambool Australia

**Keywords:** cognition, socioeconomic status, marital status, social support

## Abstract

**Background:**

Certain age‐related and medical factors have been associated with cognitive dysfunction; however, less is known regarding social determinants of health. The current study aimed to investigate associations between social determinants of health and cognitive function in a population‐based sample of men without dementia.

**Methods:**

Data were drawn from the ongoing Geelong Osteoporosis Study (*n* = 536). Cognitive function was determined using the Cog‐State Brief Battery. Area‐based socioeconomic status (SES) was determined using the Index of Relative Socioeconomic Advantage and Disadvantage, marital status by self‐report, and social support by the Multidimensional Scale of Perceived Social Support, which considers family, friends, and significant others.

**Results:**

Belonging to a higher SES group, being in a relationship (married/de‐facto) and perceived social support from a significant other and friends were each associated with better overall cognitive function. In regard to the specific cognitive domains, higher SES was associated with better psychomotor function and visual learning, being in a relationship was associated with better working memory, and perceived social support from a significant other was associated with better attention and working memory, with perceived social support from friends associated with better psychomotor function. There were no associations detected between social support from family and any of the cognitive domains.

**Conclusion:**

Higher SES, being in a relationship, and greater perceived social support from a significant other and friends were associated with better cognitive function. Further studies identifying underlying mechanisms linking social factors with cognition are needed to establish prevention strategies and enhance cognitive health.

## Abstract 2

The current study investigated the relationship between socioeconomic status, relationship status and social support and their association with both overall and specific domains of cognitive function in a population‐based sample of men without dementia.

## 1 BACKGROUND

Over the past century, lifespan has increased dramatically. Consequently, there is a rising prevalence of age‐related cognitive decline, such as mild cognitive impairment and dementia (WHO, 2023). Mild cognitive impairment is an intermediate phase between normal ageing and dementia, characterized by a modest decline in cognition greater than expected for an individual's age and education (Albert et al., 2013; Lydon et al., 2022; Petersen et al., 2001). Mild cognitive impairment affects approximately 17% of people over the age of 60 years, with prevalence increasing with increasing age (Petersen et al., 2018). Individuals with mild cognitive impairment are more likely to develop progressive cognitive decline, with an annual conversion rate to dementia of 10%–15% (Petersen et al., 2001, 2018; Petersen, 2011; Petersen, 2016). Dementia is defined as loss of cognitive abilities, being an umbrella term for a number of neurological conditions, with the most common being Alzheimer's disease. Worldwide, ∼55 million people have dementia, with nearly 10 million new cases each year (WHO, 2023). The total number of people with dementia and cognitive decline is projected to reach 78 million by 2030 and 139 million by 2050, with two‐thirds of those affected living in low‐income and middle‐income countries (WHO, 2023).

With these rapidly increasing numbers, there has been a significant focus on modifiable risk and prevention factors related to poor cognitive outcomes (Baumgart et al., 2015). 2015). Social determinants of health constitute a growing area of interest, referring to socioeconomic factors such as income, wealth and education, marital status, and social supports, such as friends and family (Braveman & Gottlieb, 2014). Interactions with social networks, utilizing different skills and participating in a range of tasks with others, have been suggested to foster greater cognitive reserve and increase cognitive function (Nie et al., 2021; Stern, 2009). For example, a study by Bennet et al. (2006) in the United States reported that older adults with Alzheimer's disease who have a larger circle of social connectedness sustained higher working and semantic memory. However, investigations into the role of social factors in relation to overall cognitive function and/or specific cognitive domains including psychomotor function, attention, working memory, and visual learning are limited, with previous studies focusing on older adults with a neurodegenerative disorder.

Thus, we aimed to investigate the relationship between socioeconomic status (SES), relationship status, social support, and their association with both overall and specific domains of cognitive function in a population‐based sample of men without dementia.

## 2 METHODS

### 2.1 Participants

Data were collected from an age‐stratified, population‐based sample of men enrolled in the Geelong Osteoporosis Study (GOS) (Bennett et al., 2006). The GOS, originally designed to investigate the epidemiology of osteoporosis, has since expanded to investigate a range of diseases including mental health. Originally, 1540 men aged between 20 and 97 years were selected at random from electoral rolls from the Barwon Statistical Division (BSD) in south‐eastern Australia. The inclusion criteria for the study included a listing on the commonwealth electoral roll as a resident of the BSD, and exclusion criteria included residents living in the area <6 months and individuals unable to provide written informed consent. Participants have returned for assessment 5‐ and 15‐year post baseline, with the current analysis utilizing data from the 15‐year follow‐up (2015−2019). Of 603 men who attended the 15‐year follow‐up at the time of writing, 67 participants did not complete the cognitive assessment, resulting in 536 eligible for inclusion in the current analyses. This study was approved by the Barwon Health Human Research Ethics Committee (00/56), and all participants gave written, informed consent.

### 2.2 Measurements

#### 2.2.1 Outcome

##### Cognitive function

Cognitive status was determined using the Cog‐State brief battery (CBB), a computer‐based neuropsychology battery for assessing specific cognitive domains for use in epidemiological studies and clinical trials (Fredrickson et al., 2010). Participants were briefed and introduced to the battery and underwent a practice trial prior to each task. The CBB has been previously validated for use in healthy populations and those with mild cognitive impairment and early dementia (Darby et al., 2002; Dingwall et al., 2009; Falleti et al., 2006; Fredrickson et al., 2010; Maruff et al., 2004). Previous studies have also reported on the efficiency, acceptability and test–retest reliability of the CBB (Fredrickson et al., 2010), deeming the CBB a good candidate for research participants with limited familiarity with computers (Fredrickson et al., 2010). The CBB comprises four computerized cognitive tasks, requiring 10–12 min for administration, and consists of (1) a simple reaction time task assessing psychomotor function (detection task [DET]). Psychomotor function refers to processing speed skills and involves the length of time it takes to process information and formulate a response (Anna‐Karin et al., 2014). (2) a choice reaction time task assessing attention (identification task [IDN]). Attention is the ability to focus and concentrate on relevant information while excluding other details (Hennawy et al., 2019). (3) a one‐back task assessing working memory (one‐back task [OBK]). Working memory is the ability to hold information in the mind while using that information (Verhaeghen et al., 2003). (4) a continuous recognition task assessing visual learning (one‐card learning task [OCL]). Visual learning is the ability to understand and work with visual information (Pal et al., 2016). Reaction time was the primary outcome measure for DET, IDN, and OBK and was calculated according to the speed of performance (log10 million seconds [lmn]), with lower scores indicating greater performance. Reaction time accuracy was the primary outcome measure for OCL and was calculated according to the accuracy of performance and reaction time, with higher scores indicating greater performance. Overall cognitive function (OCF) was generated by combining the four cognitive domains; higher scores indicated better performance, as previously described (Sui et al., 2020).

### 2.3 Exposure

*Socioeconomic status*: Area‐based SES was determined by matching residential addresses for each participant to the corresponding Australian Bureau of Statistics 2016 Census Collection District, utilizing the Socio‐Economic Index for Areas (SEIFA) value based on census data for each participant (Australian Bureau of Statistics, 2018). SEIFA values demonstrate the characteristics of participants within an area, providing a single measure to rank the level of disadvantage at the area‐level. For our study, SES was determined from the Index of Relative Socioeconomic Advantage and Disadvantage (IRSAD), which accounts for high and low income, and the type of occupation from unskilled employment to professional positions. The IRSAD scores for the participants were categorized into quintiles according to cut‐points for the study region, with quintile 1 being the most disadvantaged and quintile 5 the most advantaged.

*Marital status*: Current marital status was determined by self‐report and classified into four categories: (1) single, (2) being in a relationship (married or de‐facto), (3) divorced or separated from marriage, or (4) widowed.

*Perceived social support*: Social support was measured using the Multidimensional Scale of Perceived Social Support (MSPSS) (Zimet et al., 1988). The MSPSS is a self‐report scale measuring an individual's perception of their social support. It contains 12 items, four items for each subscale (friends, family, and significant other). There are seven possible responses to each statement scored zero to six, totaling to a maximum score of 72, with higher scores indicating greater perceived social support (Zimet et al., 1988). The MSPSS has been found to be a reliable and valid tool within different age groups and cultural backgrounds, with good to excellent internal consistency and test‐retest reliability (Bruwer et al., 2008; Clara et al., 2003; Pedersen et al., 2009; Zimet et al., 1990, 1988; Ramaswamy et al., 2009).

*Other data*: Current cigarette smoking was self‐reported. Alcohol intake was obtained from a validated food frequency questionnaire and expressed as grams per day (g/day) (Giles & Ireland, 1996). Physical activity was categorized as “active” if participants reported performing vigorous or light exercise regularly, or categorized as inactive. Weight was measured using electronic scales, height was measured using a Harpenden stadiometer, and body mass index (BMI) was calculated as weight/height^2^ (kg/m^2^).

### 2.4 Statistical analysis

Participant characteristics are summarized in Table 1 and reported as number and percentage for categorical variables and median and interquartile range (IQR) or mean and standard deviation (SD) for continuous variables. Multivariable linear regression was used to determine associations between each social factor (SES, marital status, and perceived social support) and (1) overall cognitive function (OCF), (2) psychomotor function (DET), (3) attention (IDN), (4) working memory (OBK), and (5) visual learning (OCL). Unadjusted models, age‐adjusted models, and best fit models that considered BMI, alcohol use, physical activity, and smoking, in addition to age, and retained if *p* < .05 was presented. Interactions between exposures in the best fit model were tested. Partial eta‐squared effect sizes were calculated, 0.01 indicates a small effect, 0.06 indicates a medium effect, and 0.014 indicates a large effect (Field, 2013). Analyses were undertaken using SPSS Statistics (Version 28) (IBM Corp, 2021).

**TABLE 1:** Participant characteristics (*n* = 537). Values are given as median (IQR) or *n* (%).

Age (year)	63.7 (33.1–96.4)
BMI (kg/m^2^)	27.6 (25.2–30.2)
Smoking (current)	41 (7.6%)
Physical activity (active)	399 (74.3%)
Alcohol intake (g/day)	10.7 (2.6–26.5)
SES Quintile 1 (most disadvantaged) Quintile 2 Quintile 3 Quintile 4 Quintile 5 (most advantaged)	81 (15.3%) 106 (20%) 120 (22.7%) 149 (28.2%) 73 (13.8%)
Marital status In a relationship (married/de‐facto) Single Separated/divorced Widowed	436 (81.6%) 32 (6%) 37 (6.9%) 28 (5.2%)
Perceived social support (current) Significant other Family Friends	6.5 (1−7) 6 (1−7) 5.8 (1−7)
Cognition Overall cognitive function Psychomotor function Attention Working memory Visual learning	0.1 (−0.4 to 0.5) 2.4 (2.4–2.5) 2.7 (2.6–2.7) 2.9 (2.8–2.9) 1.0 (0.9–1.0)

Abbreviation: SES, socioeconomic status.

## 3 RESULTS

A total of 536 participants were eligible for inclusion (Table 1). The median age of participants was 63 (33.1–96.4) years. Forty‐one (7.6%) participants were current smokers, and 399 (74.3%) participants were physically active. The median BMI of participants was 27.6 (IQR 25.2–30.2) kg/m^2^, and median alcohol intake was 10.7 (IQR 2.6–26.5) g/day. Over three quarters (81.6%) of the participants identified as being in a relationship at the time of assessment. The median overall cognitive function was 0.1 (−0.4 to 0.5) lmn, psychomotor function was 2.4 (2.4–2.5) lmn, attention was 2.7 (2.6–2.7) lmn, working memory was 2.9 (2.8–2.9) lmn and visual learning was 1.0 (0.9–1.0) lmn.

### 3.1 Socioeconomic status

Unadjusted and age‐adjusted data are presented in Table 2, with the lowest SES group (quintile 1, most disadvantaged) held as referent. Compared to the most disadvantaged, increasing advantage was associated with better overall cognitive function, psychomotor function, and visual learning. These patterns persisted after adjustment for age, albeit with some losing significance. BMI, smoking, alcohol intake, and physical activity did not contribute to the models. The effect size for all relationships ranged from 0.004 to 0.016. No associations were evident between SES and attention or working memory before or after adjustment for age (all *p* > .05).

**TABLE 2:** Unadjusted and age adjusted multiple regression models showing associations between socioeconomic status (SES), relationship status and social support, and cognitive function.

	Overall cognitive function				Psychomotor function				Attention				Working memory				Visual learning			
	*b*	SE	*η* ^2^	*p*	*b*	SE	*η* ^2^	*p*	*b*	SE	*η* ^2^	*p*	*b*	SE	*η* ^2^	*p*	*b*	SE	*η* ^2^	*p*
Unadjusted																				
SES																				
Quintile 1 Quintile 2 Quintile 3 Quintile 4 Quintile 5	‐ 0.183 0.232 0.239 0.358	‐ 0.111 0.108 0.104 0.121	‐ 0.005 0.009 0.010 0.017	‐ 0.101 **0.033** **0.022** **0.003**	‐ −0.011 −0.039 −0.042 −0.059	‐ 0.016 0.015 0.015 0.017	‐ 0.001 0.012 0.015 0.022	‐ 0.472 **0.012** **0.004** **< 0.001**	‐ −0.007 −0.012 −0.014 −0.018	‐ 0.011 0.011 0.011 0.012	‐ 0.001 0.002 0.003 0.004	‐ 0.528 0.273 0.188 0.134	‐ −0.010 −0.015 −0.008 −0.026	‐ 0.017 0.016 0.015 0.018	‐ 0.001 0.002 0.001 0.004	‐ 0.561 0.363 0.588 0.153	‐ 0.039 0.028 0.030 0.041	‐ 0.015 0.014 0.014 0.016	‐ 0.013 0.007 0.009 0.012	‐ **0.009** **0.056** **0.030** **0.012**
Marital status Single Married/de‐facto Separated/divorce Widowed	‐ 0.073 0.016 −0.770	‐ 0.133 0.175 0.191	‐ 0.001 < 0.001 0.030	‐ 0.582 0.927 **< 0.001**	‐ 0.001 0.007 0.088	‐ 0.019 0.026 0.027	‐ < 0.001 < 0.001 0.019	‐ 0.994 0.781 **0.001**	‐ −0.004 −0.001 0.080	‐ 0.014 0.018 0.019	‐ < 0.001 < 0.001 0.032	‐ 0.749 0.947 **< 0.001**	‐ −0.020 −0.016 0.082	‐ 0.020 0.026 0.029	‐ 0.002 0.001 0.015	‐ 0.316 0.546 **0.004**	‐ 0.005 −0.003 −0.046	‐ 0.018 0.024 0.026	‐ < 0.001 < 0.001 0.006	‐ 0.779 0.913 0.082
Social support																				
Other Family Friends	0.039 0.005 0.027	0.023 0.024 0.023	0.006 0.001 0.003	0.088 0.821 0.245	‐0.005 −0.001 −0.006	0.003 0.003 0.003	0.004 0.001 0.006	0.125 0.792 0.065	‐0.005 −0.001 −0.002	0.002 0.002 0.002	0.008 0.001 0.002	**0.042** 0.825 0.302	‐0.005 −0.001 −0.003	0.003 0.003 0.003	0.003 0.001 0.001	0.175 0.762 0.391	0.001 −0.001 −0.001	0.003 0.003 0.003	0.001 0.001 0.001	0.852 0.825 0.679
Age Adjusted																				
SES																				
Quintile 1 Quintile 2 Quintile 3 Quintile 4 Quintile 5	‐ 0.190 0.175 0.163 0.259	‐ 0.094 0.091 0.088 0.102	‐ 0.008 0.007 0.007 0.012	‐ **0.043** 0.055 0.063 **0.011**	‐ −0.013 −0.034 −0.035 −0.039	‐ 0.015 0.014 0.014 0.016	‐ 0.002 0.011 0.012 0.018	‐ 0.367 **0.018** **0.012** **0.002**	‐ −0.008 −0.007 −0.007 −0.010	‐ 0.010 0.010 0.009 0.011	‐ 0.001 0.001 0.001 0.001	‐ 0.408 0.445 0.445 0.384	‐ −0.011 −0.007 0.001 −0.013	‐ 0.015 0.014 0.014 0.016	‐ 0.001 0.001 0.001 0.001	‐ 0.468 0.603 0.935 0.415	‐ 0.040 0.024 0.024 0.033	‐ 0.014 0.014 0.013 0.015	‐ 0.015 0.006 0.006 0.009	‐ **0.005** 0.084 0.073 **0.033**
Marital status Single Married/de‐facto Separated/divorce Widowed	‐ 0.305 0.169 0.032	‐ 0.115 0.151 0.174	‐ 0.013 0.002 < 0.001	‐ **0.008** 0.262 0.856	‐ −0.023 −0.008 0.007	‐ 0.018 0.024 0.027	‐ 0.003 < 0.001 < 0.001	‐ 0.203 0.734 0.805	‐ −0.024 −0.014 0.013	‐ 0.012 0.016 0.019	‐ 0.007 0.001 0.001	‐ 0.060 0.402 0.487	‐ −0.050 −0.036 −0.023	‐ 0.018 0.023 0.027	‐ 0.015 0.004 0.001	‐ 0.005 0.128 0.400	‐ 0.025 0.010 0.022	‐ 0.018 0.023 0.026	‐ 0.004 < 0.001 0.001	‐ 0.160 0.657 0.396
Social support Other Family Friends	0.057 0.032 0.045	0.019 0.020 0.020	0.016 0.005 0.010	**0.003** 0.117 **0.023**	−0.007 −0.004 −0.008	0.003 0.003 0.003	0.010 0.001 0.013	**0.023** 0.214 **0.008**	−0.006 −0.003 −0.004	0.002 0.002 0.002	0.016 0.003 0.007	**0.004** 0.218 0.062	−0.007 −0.004 −0.005	0.003 0.003 0.003	0.009 0.003 0.005	**0.028** 0.197 0.101	0.002 0.002 0.001	0.003 0.003 0.003	0.001 0.001 0.001	0.471 0.554 0.901
Best model^*^																				
SES																				
Quintile 1 Quintile 2 Quintile 3 Quintile 4 Quintile 5	‐ 0.178 0.136 0.134 0.202	‐ 0.093 0.092 0.088 0.103	‐ 0.007 0.004 0.004 0.007	‐ 0.058 0.140 0.129 0.051	‐ −0.011 −0.027 −0.030 −0.040	‐ 0.015 0.014 0.014 0.016	‐ 0.001 0.007 0.009 0.012	‐ 0.463 0.058 **0.033** **0.014**	‐ −0.007 −0.003 −0.004 −0.004	‐ 0.010 0.010 0.009 0.011	‐ 0.001 0.001 0.001 0.001	‐ 0.477 0.734 0.676 0.724	‐ −0.009 −0.003 0.004 −0.008	‐ 0.015 0.014 0.014 0.016	‐ 0.001 0.001 0.001 0.001	‐ 0.529 0.812 0.747 0.631	‐ 0.040 0.023 0.024 0.030	‐ 0.014 0.014 0.013 0.016	‐ 0.016 0.005 0.006 0.007	‐ **0.004** 0.099 0.076 0.053
Marital status Single Married/de‐facto Separated/divorce Widowed	‐ 0.262 0.142 0.020	‐ 0.115 0.150 0.173	‐ 0.010 0.002 < 0.001	‐ **0.021** 0.344 0.910	‐ −0.017 −0.005 0.009	‐ 0.018 0.024 0.027	‐ 0.002 < 0.001 < 0.001	‐ 0.367 0.845 0.738	‐ −0.020 −0.011 0.015	‐ 0.012 0.016 0.018	‐ 0.005 0.001 0.001	‐ 0.113 0.480 0.432	‐ −0.048 −0.033 −0.022	‐ 0.018 0.023 0.027	‐ 0.013 0.004 0.001	‐ **0.008** 0.159 0.423	‐ 0.022 0.008 0.024	‐ 0.018 0.023 0.027	‐ 0.003 < 0.001 0.002	‐ 0.208 0.735 0.361
Social support Other Family Friends	0.052 0.031 0.039	0.019 0.020 0.020	0.014 0.005 0.008	**0.008** 0.116 **0.046**	−0.006 −0.003 −0.007	0.003 0.003 0.003	0.006 0.002 0.010	0.073 0.303 **0.024**	−0.006 −0.003 −0.004	0.002 0.002 0.002	0.013 0.003 0.005	**0.008** 0.195 0.098	−0.007 −0.005 −0.005	0.003 0.003 0.003	0.009 0.004 0.005	**0.030** 0.142 0.120	0.002 0.002 0.001	0.003 0.003 0.003	0.001 0.001 0.001	0.526 0.569 0.997

*Note*: *η* ^2^ = partial eta. 0.01 indicates a small effect, 0.06 indicates a medium effect, and 0.014 indicates a large effect. Bold values are significant.

* Best model = age, BMI, smoking, alcohol intake and physical activity.

### 3.2 Marital status

In unadjusted models, married/de‐facto was associated with better overall cognitive function and working memory, but not psychomotor function, attention, and visual learning compared to those who are single (Table 2). These associations persisted after adjustment for age, BMI, smoking, alcohol intake, and physical activity. The effect size for all relationships ranged from 0.010 to 0.013.

### 3.3 Perceived social support

Unadjusted and age‐adjusted results are presented in Table 2.

#### 3.3.1 Significant other

In unadjusted models, support from a significant other was associated with better attention, but not overall cognitive function, psychomotor function, working memory, or visual learning. Following adjustment for age, support from a significant other was associated with better overall cognitive function, psychomotor function, attention, and working memory, but not visual learning. BMI, smoking, alcohol intake, and physical activity did not contribute to the models. The effect size for all relationships ranged from 0.009 to 0.013.

#### 3.3.2 Family

Before and after adjustment for age, support from family was not associated with any of the cognitive domains (psychomotor function, attention, working memory, and visual learning, all *p* > .05).

#### 3.3.3 Friends

In unadjusted models, perceived social support from friends was not associated with any of the cognitive domains (psychomotor function, attention, working memory, and visual learning, all *p* > .05). In age‐adjusted models, social support from friends was associated with better overall cognitive function and psychomotor function, but not attention, working memory, and visual learning compared to low social support from friends. These relationships persisted after further adjustment for BMI, smoking, alcohol intake, and physical activity. The effect size for all relationships ranged from 0.008 to 0.010.

## 4 DISCUSSION

In this cross‐sectional, population‐based study of men, higher SES, being in a relationship, and perceived social support from a significant other and friends were associated with better overall cognitive function. In regard to specific domains, higher SES was associated with better psychomotor function and visual learning. Being married or in a de‐facto relationship was associated with better working memory. Perceived social support from a significant other was associated with better attention, and working memory and perceived social support from friends were associated with better psychomotor function. No associations were evident between being separated/divorced, widowed, or perceived social support from family with any of the cognitive domains.

Similar to our results, albeit in older populations, studies have reported low SES to be associated with poorer cognitive health (Dalstra et al., 2005; Petersen et al., 2021; Wallis et al., 2002; Z. Zhang et al., 2022). A recent cross‐sectional study by Z. Zhang et al. (2022) reported greater cognitive impairment measured by The Montreal Cognitive Assessment among people aged 65 years and older with lower SES. Moreover, a large cross‐sectional study of elderly patients (*n* = 1420) found that socioeconomic deprivation was associated with lower cognitive function utilizing the Mini‐Mental State Examination (Park et al., 2017). Furthermore, Qian et al. (2014) found in a clinical sample of older adults (>65 years) that those identified as having lower SES were less likely to seek help or postponed seeking help for their cognitive health compared to those with higher SES. When investigating specific domains of cognition, we found higher SES was related to psychomotor function and visual learning, suggesting better problem‐solving skills and processing capacity.

Supporting a small body of literature in elderly populations (Bae et al., 2015; Chen et al., 2021; Feng et al., 2014; Hui et al., 2020), single individuals were found to be at a greater risk of cognitive decline than those in a relationship. A previous cross‐sectional study of elderly Chinese residents aged over 65 years (*n* = 19,276) found that being divorced, separated, widowed, or single was associated with a greater risk of cognitive decline and dementia compared to residents in a relationship (Chen et al., 2021).

Consistent with our results, a growing body of evidence indicates a positive association between social support and global cognitive function among the elderly (Holtzman et al., 2004; Krueger et al., 2009; Yeh & Liu, 2003). Yeh and Liu (2003) found that higher cognitive function assessed by the Short Portable Mental Status Questionnaire was associated with increased perceived social support in a large sample (*n* = 4993) of adults aged >65 years. Interestingly, in the current study perceived social support from family members was not associated with cognitive function, with this result being similar to others (Kuiper et al., 2016; Pillemer & Holtzer, 2016). A cross‐sectional study by Pillemer and Holtzer (2016) of community‐residing older adults reported that perceived emotional and informational support and positive social interactions were all associated with better cognition. Additionally, previous evidence suggests older people who are more socially supported have higher levels of cognitive function compared to less socially supported individuals (Barnes et al., 2004; Bassuk et al., 1999; Holtzman et al., 2004; Krueger et al., 2009; Yeh & Liu, 2003; Zunzunegui et al., 2003). In a prospective cohort study that followed 1203 individuals without dementia aged 75 and over, it was reported that people with a large social network were at reduced risk of dementia (Fratiglioni et al., 2000). While some studies reported that a larger social network is better for cognitive function (Crooks et al., 2008; Gureje et al., 2011; Scarmeas et al., 2001), others reported the size of social contacts is not strongly associated with cognition in old age (Hughes et al., 2008; ). This may be due to the satisfaction of social supports being more important than the number of social connections (Glei et al., 2005; Krueger et al., 2009). Therefore, it is suggested that social contacts and perceived social support from family, friends, and acquaintances are important for fulfilment of different social needs (Yeh & Liu, 2003).

Several mechanisms underlying the link between social determinants of health and cognition have been suggested. First, sustaining social supports requires cognitive strategies which are likely to help build cognitive reserve through cognitive exercise and stimulation, shown to benefit memory and executive function (Cohen, 2016; Luethi et al., 2008; Scarmeas et al., 2006). Additionally, social determinants of health may act as a buffer against stressful situations and promote healthy lifestyle behaviors associated with fluid reasoning, attention, and psychomotor skills (Bae et al., 2015). It has also been suggested that people who accumulate more wealth live in better environments and less stressful conditions, further contributing to better cognitive health in later life (Z. X. Zhang et al., 2009; Z. Zhang et al., 2008). Moreover, people with higher SES often have a higher education and are in professions that require a high level of cognitive functioning (Qian et al., 2014). Thus, individuals with more cognition‐demanding jobs may be able to perceive their own cognitive changes, leading them to seek help earlier, lessening cognitive decline (Qian et al., 2014). Furthermore, the marital resource model suggests being married is associated with unique social, psychological, and economic resources that are not typically obtained from other types of relationships, and this in turn promotes cognitive health (Waite & Gallagher, 2000). In addition, the stress model emphasizes that negative aspects of martial disruption, such as divorce and widowhood, create stress and undermine health, and may directly affect overall cognition and domains of working memory, psychomotor skills, and visual spatial abilities (Hughes et al., 2008; Williams & Umberson, 2004; Wilson et al., 2015).

This study has several strengths. First, we examined cognition in a large population‐based study of unselected men without dementia spanning the adult age range. Given participants spanned the full adult age range, age was tested as a confounder and/or effect modifier. Second was our ability to adjust for potential confounders that may explain the relationships explored. Third, a comprehensive cognitive test battery was used to assess cognition, which allowed the investigation of specific cognitive domains. However, our study has some limitations. The study included men only; accordingly, interpretation may not be generalizable to women, and therefore future research may consider collecting comparable data. Furthermore, as the CBB is a computerized battery, it does need to be acknowledged that a lack of familiarity with computers may influence performance. Additionally, as our study utilized participants from the general population, those who participated were more likely to have healthier profiles compared to those who did not.

In conclusion, higher SES, being in a relationship, and greater perceived social support from a significant other and friends were associated with better cognitive function in this population‐based sample of men. Such evidence highlights social determinants of health to be associated with cognition in adulthood. Identifying mechanisms and interactions between these exposures on cognitive function is vital for timely treatment and future prevention strategies, as well as targeting those who may benefit more from intervention.

## AUTHOR CONTRIBUTIONS

Kayla B. Corney, Lana J. Williams, and Julie A. Pasco planned and designed the study. Mohammadreza Mohebbi provided feedback for the statistical analyses. Kayla B. Corney and Amanda L. Stuart performed the analyses. Kayla B. Corney, Amanda L. Stuart, Lana J. Williams, and Julie A. Pasco contributed to the interpretation of the data. Kayla B. Corney took primary responsibility for writing the manuscript. Lana J. Williams, Julie A. Pasco, Amanda L. Stuart, Bianca E. Kavanagh, Mohammadreza Mohebbi, and Sophia X. Sui provided critical revisions to the article. All authors read and approved the final manuscript to be published.

## CONFLICT OF INTEREST STATEMENT

The authors declare no conflict of interest.

### PEER REVIEW

The peer review history for this article is available at https://publons.com/publon/10.1002/brb3.3235.

## Data Availability

Study data were collected and managed using Redcap electronic data capture tool hosted and managed by Research Technology Services at UNSW Sydney. The authors confirm that the data supporting the findings of this study are available within the article.

## References

[brb33235-bib-0001] Australian Bureau of Statistics . (2018). Census of population and housing: Estimating homelessness, 2016—External site opens in new window. ABS, Australian Government.

[brb33235-bib-0002] Albert, M. S. , Dekosky, S. T. , Dickson, D. , Dubois, B. , Feldman, H. H. , Fox, N. C. , Gamst, A. , Holtzman, D. M. , Jagust, W. J. , Petersen, R. C. , Snyder, P. J. , Carrillo, M. C. , Thies, B. , & Phelps, C. H. (2013). The diagnosis of mild cognitive impairment due to Alzheimer's disease: Recommendations from the National Institute on Aging‐Alzheimer's Association workgroups on diagnostic guidelines for Alzheimer's disease. Focus, 11, 96–106. 10.1176/appi.focus.11.1.96 PMC3312027 21514249

[brb33235-bib-0003] Bae, J. B. , Kim, Y. J. , Han, J. W. , Kim, T. H. , Park, J. H. , Lee, S. B. , Lee, J. J. , Jeong, H. G. , Kim, J. L. , Jhoo, J. H. , Yoon, J. C. , & Kim, K. W (2015). Incidence of and risk factors for Alzheimer's disease and mild cognitive impairment in Korean elderly. Dementia and Geriatric Cognitive Disorders, 39, 105–115. 10.1159/000366555 25401488

[brb33235-bib-0004] Barnes, L. L. , Mendes De Leon, C. F. , Wilson, R. S. , Bienias, J. L. , & Evans, D. A. (2004). Social resources and cognitive decline in a population of older African Americans and Whites. Neurology, 63, 2322–2326. 10.1212/01.WNL.0000147473.04043.B3 15623694

[brb33235-bib-0005] Bassuk, S. S. , Glass, T. A. , & Berkman, L. F. (1999). Social disengagement and incident cognitive decline in community‐dwelling elderly persons. Annals of Internal Medicine, 131, 165–173. 10.7326/0003-4819-131-3-199908030-00002 10428732

[brb33235-bib-0006] Baumgart, M. , Snyder, H. M. , Carrillo, M. C. , Fazio, S. , Kim, H. , & Johns, H. (2015). Summary of the evidence on modifiable risk factors for cognitive decline and dementia: A population‐based perspective. Alzheimers & Dementia, 11(6), 718–726. 10.1016/j.jalz.2015.05.016 26045020

[brb33235-bib-0007] Bennett, D. A. , Schneider, J. A. , Tang, Y. , Arnold, S. E. , & Wilson, R. S. (2006). The effect of social networks on the relation between Alzheimer's disease pathology and level of cognitive function in old people: A longitudinal cohort study. Lancet Neurology, 5, 406–412. 10.1016/S1474-4422(06)70417-3 16632311

[brb33235-bib-0008] Braveman, P. , & Gottlieb, L. (2014). The social determinants of health: It's time to consider the causes of the causes. Public Health Reports (Washington, D.C. : 1974), 129(2), 19–31. 10.1177/00333549141291S206 PMC3863696 24385661

[brb33235-bib-0009] Bruwer, B. , Emsley, R. , Kidd, M. , Lochner, C. , & Seedat, S. (2008). Psychometric properties of the Multidimensional Scale of Perceived Social Support in youth. Comprehensive Psychiatry, 49, 195–201. 10.1016/j.comppsych.2007.09.002 18243894

[brb33235-bib-0010] Chen, Z.‐C. , Wu, H. , Wang, X.‐D. , Zhou, X. , Zhou, W. , Zhu, L. , Wang, T. , Tu, J. , Bao, H. , & Cheng, X. (2021). Association between marital status and cognitive impairment based on a cross‐sectional study in China. International Journal of Geriatric Psychiatry, 22, 504. 10.1002/gps.5649 34729814

[brb33235-bib-0011] Clara, I. P. , Cox, B. J. , Enns, M. W. , Murray, L. T. , & Torgrudc, L. J. (2003). Confirmatory factor analysis of the multidimensional scale of perceived social support in clinically distressed and student samples. Journal of Personality Assessment, 81, 265–270. 10.1207/S15327752JPA8103_09 14638451

[brb33235-bib-0013] Crooks, V. C. , Lubben, J. , Petitti, D. B. , Little, D. , & Chiu, V. (2008). Social network, cognitive function, and dementia incidence among elderly women. American Journal of Public Health, 98, 1221–1227. 10.2105/AJPH.2007.115923 18511731 PMC2424087

[brb33235-bib-0014] Dalstra, J. , Kunst, A. , Borrell, C. , Breeze, E. , Cambois, E. , Costa, G. , Geurts, J. , Lahelma, E. , Van Oyen, H. , Rasmussen, N. , Regidor, E. , Spadea, T. , & Mackenbach, J. (2005). Socioeconomic differences in the prevalence of common chronic diseases: An overview of eight European countries. International Journal of Epidemiology, 34, 316–326. 10.1093/ije/dyh386 15737978

[brb33235-bib-0015] Darby, D. , Maruff, P. , Collie, A. , & Mcstephen, M. (2002). Mild cognitive impairment can be detected by multiple assessments in a single day. Neurology, 59(7), 1042–1046. 10.1212/WNL.59.7.1042 12370459

[brb33235-bib-0016] Dingwall, K. M. , Lewis, M. S. , Maruff, P. , & Cairney, S. (2009). Reliability of repeated cognitive testing in healthy Indigenous Australian adolescents. Australian Psychologist, 44(4), 224–234. 10.1080/00050060903136839

[brb33235-bib-0017] Falleti, M. G. , Maruff, P. , Collie, A. , & Darby, D. G. (2006). Practice effects associated with the repeated assessment of cognitive function using the CogState battery at 10‐minute, one week and one month test‐retest intervals. Journal of Clinical and Experimental Neuropsychology, 28(7), 1095–1112. 10.1080/13803390500205718 16840238

[brb33235-bib-0018] Feng, L. , Ng, X.‐T. , Yap, P. , Li, J. , Lee, T.‐S. , Håkansson, K. , Kua, E.‐H. , & Ng, T.‐P. (2014). Marital status and cognitive impairment among community‐dwelling Chinese older adults: The role of gender and social engagement. Dementia and Geriatric Cognitive Disorders Extra, 4(3), 375–384. 10.1159/000358584 25473404 PMC4241637

[brb33235-bib-0019] Field, A. (2013). Discovering Statistics Using IBM SPSS Statistics. SAGE.

[brb33235-bib-0020] Fratiglioni, L. , Wang, H.‐X. , Ericsson, K. , Maytan, M. , & Winblad, B. (2000). Influence of social network on occurrence of dementia: A community‐based longitudinal study. Lancet, 355, 1315–1319. 10.1016/S0140-6736(00)02113-9 10776744

[brb33235-bib-0021] Fredrickson, J. , Maruff, P. , Woodward, M. , Moore, L. , Fredrickson, A. , Sach, J. , & Darby, D. (2010). Evaluation of the usability of a brief computerized cognitive screening test in older people for epidemiological studies. Neuroepidemiology, 34(2), 65–75. 10.1159/000264823 20016215

[brb33235-bib-0022] Giles, G. G. , & Ireland, P. D (1996). Dietary questionnaire for epidemiological studies (version 2). The Cancer Council Victoria.

[brb33235-bib-0023] Glei, D. A. , Landau, D. A. , Goldman, N. , Chuang, Y.‐L. , Rodríguez, G. , & Weinstein, M. (2005). Participating in social activities helps preserve cognitive function: An analysis of a longitudinal, population‐based study of the elderly. International Journal of Epidemiology, 34, 864–871. 10.1093/ije/dyi049 15764689

[brb33235-bib-0024] Gureje, O. , Ogunniyi, A. , Kola, L. , & Abiona, T. (2011). Incidence of and risk factors for dementia in the Ibadan Study of Aging. Journal of the American Geriatrics Society, 59, 869–874. 10.1111/j.1532-5415.2011.03374.x 21568957 PMC3173843

[brb33235-bib-0025] Harris, P. A. , Taylor, R. , Thielke, R. , Payne, J. , Gonzalez, N. , & Conde, J. G. (2009). Research electronic data capture (REDCap)—A metadata‐driven methodology and workflow process for providing translational research informatics support. Journal of Biomedical Informatics, 42(2), 377–381. 10.1016/j.jbi.2008.08.010 18929686 PMC2700030

[brb33235-bib-0026] Hennawy, M. , Sabovich, S. , Liu, C. S. , Herrmann, N. , & Lanctôt, K. L. (2019). Sleep and attention in Alzheimer's disease. The Yale Journal of Biology and Medicine, 92(1), 53–61. 30923473 PMC6430169

[brb33235-bib-0027] Holtzman, R. E. , Rebok, G. W. , Saczynski, J. S. , Kouzis, A. C. , Wilcox Doyle, K. , & Eaton, W. W. (2004). Social network characteristics and cognition in middle‐aged and older adults. Journal of Gerontology: Psychological Sciences, 59, P278–P284. 10.1093/geronb/59.6.P278 15576855

[brb33235-bib-0028] Hughes, T. F. , Andel, R. , Small, B. J. , Borenstein, A. R. , & Mortimer, J. A. (2008). The association between social resources and cognitive change in older adults: Evidence from the Charlotte County healthy aging study. Journals of Gerontology. Series B, Psychological Sciences and Social Sciences, 63(4), P241–P244. 10.1093/geronb/63.4.P241 18689766

[brb33235-bib-0029] Hui, L. , Zhenmei, Z. , Seung‐won, C. , & Kenneth, M. L. (2020). Marital status and dementia: Evidence from the health and retirement study. The Journals of Gerontology: Series B, 75(8), 1783–1795. 10.1093/geronb/gbz087 PMC7489107 31251349

[brb33235-bib-0030] IBM Corp . (2021). IBM SPSS Statistics for Windows, Version 28.0. IBM Corp.

[brb33235-bib-0031] Krueger, K. R. , Wilson, R. S. , Kamenetsky, J. M. , Barnes, L. L. , Bienias, J. L. , & Bennett, D. A. (2009). Social engagement and cognitive function in old age. Experimental Aging Research, 35(1), 45–60. 10.1080/03610730802545028 19173101 PMC2758920

[brb33235-bib-0032] Kuiper, J. S. , Zuidersma, M. , Zuidema, S. U. , Burgerhof, J. G. M. , Stolk, R. P. , Voshaar, O. , R. C. , & Smidt, N. (2016). Social relationships and cognitive decline: A systematic review and meta‐analysis of longitudinal cohort studies. International Journal of Epidemiology, 45, 1169–1206. 27272181 10.1093/ije/dyw089

[brb33235-bib-0033] Luethi, M. (2008). Stress effects on working memory, explicit memory, and implicit memory for neutral and emotional stimuli in healthy men. Frontiers in Behavioral Neuroscience, 2, 5. 10.3389/neuro.08.005.2008 19169362 PMC2628592

[brb33235-bib-0034] Lydon, E. A. , Nguyen, L. T. , Nie, Q. , Rogers, W. A. , & Mudar, R. A. (2022). An integrative framework to guide social engagement interventions and technology design for persons with mild cognitive impairment. Frontiers in Public Health, 9, 750340. https://www.frontiersin.org/articles/10.3389/fpubh.2021.750340 35096730 10.3389/fpubh.2021.750340 PMC8795670

[brb33235-bib-0035] Maruff, P. , Collie, A. , Darby, D. , Weaver‐Cargin, J. , Masters, C. , & Currie, J. (2004). Subtle memory decline over 12 months in mild cognitive impairment. Dementia and Geriatric Cognitive Disorders, 18, 342–348. 10.1159/000080229 15316183

[brb33235-bib-0036] Nie, Y. , Richards, M. , Kubinova, R. , Titarenko, A. , Malyutina, S. , Kozela, M. , Pajak, A. , Bobak, M. , & Ruiz, M. (2021). Social networks and cognitive function in older adults: Findings from the HAPIEE study. BMC Geriatrics, 21, 570. 10.1186/s12877-021-02531-0 34663241 PMC8524850

[brb33235-bib-0037] Pal, A. , Biswas, A. , Pandit, A. , Roy, A. , Guin, D. , Gangopadhyay, G. , & Senapati, A. (2016). Study of visuospatial skill in patients with dementia. Annals of Indian Academy of Neurology, 19(1), 83–88. 10.4103/0972-2327.168636 27011635 PMC4782559

[brb33235-bib-0038] Park, M. H. , Smith, S. C. , Neuburger, J. , Chrysanthaki, T. , Hendriks, A. A. J. , & Black, N. (2017). Sociodemographic characteristics, cognitive function, and health‐related quality of life of patients referred to memory assessment services in England. Alzheimer Disease & Associated Disorders, 31(2), 159–167. 27819844 10.1097/WAD.0000000000000166

[brb33235-bib-0039] Pasco, J. A. , Nicholson, G. C. , & Kotowicz, M. A. (2012). Cohort profile: Geelong Osteoporosis Study. International Journal of Epidemiology, 41(6), 1565–1575. 10.1093/ije/dyr148 23283714

[brb33235-bib-0040] Pedersen, S. S. , Spinder, H. , Erdman, R. A. M. , & Denollet, J. (2009). Poor perceived social support in implantable cardioverter defibrillator (ICD) patients and their partners: Cross‐validation of the multidimensional scale of perceived social support. Psychosomatics, 50, 461–467. 10.1016/S0033-3182(09)70838-2 19855031

[brb33235-bib-0041] Petersen, J. D. , Wehberg, S. , Packness, A. , Svensson, N. H. , Hyldig, N. , Raunsgaard, S. , Andersen, M. K. , Ryg, J. , Mercer, S. W. , Søndergaard, J. , & Waldorff, F. B. (2021). Association of socioeconomic status with dementia diagnosis among older adults in Denmark. JAMA Network Open, 4(5), e2110432. 10.1001/jamanetworkopen.2021.10432 34003271 PMC8132141

[brb33235-bib-0042] Petersen, R. C. (2011). Clinical practice. Mild cognitive impairment. New England Journal of Medicine, 364, 2227–2234. 10.1056/NEJMcp0910237 21651394

[brb33235-bib-0043] Petersen, R. C. (2016). Mild cognitive impairment. Continuum: Lifelong Learning in Neurology, 22, 404. 10.1212/CON.0000000000000313 27042901 PMC5390929

[brb33235-bib-0044] Petersen, R. C. , Doody, R. , Kurz, A. , Mohs, R. C. , Morris, J. C. , Rabins, P. V. , Ritchie, K. , Rossor, M. , Thal, L. , & Winblad, B. (2001). Current concepts in mild cognitive impairment. Archives of Neurology, 58, 1985–1992. 10.1001/archneur.58.12.1985 11735772

[brb33235-bib-0045] Petersen, R. C. , Lopez, O. , Armstrong, M. J. , Getchius, T. S. D. , Ganguli, M. , Gloss, D. , Gronseth, G. S. , Marson, D. , Pringsheim, T. , Day, G. S. , Sager, M. , Stevens, J. , & Rae‐Grant, A. (2018). Practice guideline update summary: Mild cognitive impairment: Report of the guideline development, dissemination, and implementation subcommittee of the. American Academy of Neurology, 90, 126–135. 10.1212/WNL.0000000000004826 29282327 PMC5772157

[brb33235-bib-0046] Pillemer, S. C. , & Holtzer, R. (2016). The differential relationships of dimensions of perceived social support with cognitive function among older adults. Aging & Mental Health, 20(7), 727–735. 25902848 10.1080/13607863.2015.1033683 PMC4618790

[brb33235-bib-0047] Qian, W. , Schweizer, T. A. , & Fischer, C. E. (2014). Impact of socioeconomic status on initial clinical presentation to a memory disorders clinic. International Psychogeriatrics, 26(4), 597–603. 10.1017/S1041610213002299 24331159

[brb33235-bib-0048] Ramaswamy, V. , Aroian, K. J. , & Templin, T. (2009). Adaptation and psychometric evaluation of the multidimensional scale of perceived social support for Arab American adolescents. American Journal of Community Psychology, 43, 49–56. 10.1007/s10464-008-9220-x 19160040

[brb33235-bib-0049] Scarmeas, N. , Levy, G. , Tang, M.‐X. , Manly, J. , & Stern, Y. (2001). Influence of leisure activity on the incidence of Alzheimer's disease. Neurology, 57, 2236–2242. 10.1212/WNL.57.12.2236 11756603 PMC3025284

[brb33235-bib-0050] Scarmeas, N. , Stern, Y. , Tang, M.‐X. , Mayeux, R. , & Luchsinger, J. A. (2006). Mediterranean diet and risk for Alzheimer's disease. Annals of Neurology, 59, 912–921. 10.1002/ana.20854 16622828 PMC3024594

[brb33235-bib-0051] Stern, Y. (2009). Cognitive reserve. Neuropsychologia, 47, 2015–2028. 10.1016/j.neuropsychologia.2009.03.004 19467352 PMC2739591

[brb33235-bib-0052] Sui, S. X. , Holloway‐Kew, K. L. , Hyde, N. K. , Williams, L. J. , Leach, S. , & Pasco, J. A. (2020). Muscle strength and gait speed rather than lean mass are better indicators for poor cognitive function in older men. Scientific Reports, 10(1), 10367. 10.1038/s41598-020-67251-8 32587294 PMC7316855

[brb33235-bib-0053] WHO . (2023). Dementia . https://www.who.int/news‐room/fact‐sheets/detail/dementia

[brb33235-bib-0054] Verhaegen, P. , Borchelt, M. , & Smith, J. (2003). Relation between cardiovascular and metabolic disease and cognition in very old age: Cross‐sectional and longitudinal findings from the berlin aging study. Health Psychology, 22(6), 559–569. 10.1037/0278-6133.22.6.559 14640852

[brb33235-bib-0055] Waite, L. , & Gallagher, M. (2000). The case for marriage: Why married people are happier, healthier, and better off financially. Crown.

[brb33235-bib-0056] Wallis, E. J. (2002). Cardiovascular and coronary risk estimation in hypertension management. Heart, 88, 306–312. 12181232 10.1136/heart.88.3.306 PMC1767334

[brb33235-bib-0057] Welmer, A.‐N. , Rizzuto, D. , Qui, C. , Caracciolo, B. , & Laukka, E. J. (2014). Walking speed, processing speed, and dementia: A population‐based longitudinal study. The Journals of Gerontology: Series A, 69(12), 1503–1510. 10.1093/gerona/glu047 24706441

[brb33235-bib-0058] Williams, K. , & Umberson, D. (2004). Marital status, marital transitions, and health: A gendered life course perspective. Journal of Health and Social Behavior, 45(1), 81–98. 10.1177/002214650404500106 15179909 PMC4061612

[brb33235-bib-0059] Wilson, R. S. , Boyle, P. A. , James, B. D. , Leurgans, S. E. , Buchman, A. S. , & Bennett, D. A. (2015). Negative social interactions and risk of mild cognitive impairment in old age. Neuropsychology, 29(4), 561–570. 10.1037/neu0000154 25495828 PMC4468039

[brb33235-bib-0060] Yeh, S.‐C. J. , & Liu, Y.‐Y. (2003). Influence of social support on cognitive function in the elderly. BMC Health Service Research, 3, 9. 10.1186/1472-6963-3-9 PMC161808 12775218

[brb33235-bib-0061] Zhang, Z. , Gu, D. , & Hayward, M. D. (2008). Early life influences on cognitive impairment among oldest old Chinese. The Journals of Gerontology. Series B, Psychological Sciences and Social Sciences, 63(1), S25–S33. 10.1093/geronb/63.1.S25 18332198

[brb33235-bib-0062] Zhang, Z. , Zhao, Y. , & Bian, Y. (2022). A role of socioeconomic status in cognitive impairment among older adults in Macau: A decomposition approach. Frontiers in Aging Neuroscience, 14, 804307. 35211006 10.3389/fnagi.2022.804307 PMC8862725

[brb33235-bib-0063] Zhang, Z. X. , Plassman, B. L. , Xu, Q. , Zahner, G. E. P. , Wu, B. , Gai, M. Y. , Wen, H. B. , Chen, X. , Gao, S. , Hu, D. , Xiao, X. H. , Shen, Y. , Liu, A. M. , & Xu, T. (2009). Lifespan influences on mid‐ to late‐life cognitive function in a Chinese birth cohort. Neurology, 73(3), 186–194. 10.1212/WNL.0b013e3181ae7c90 19620606 PMC2843580

[brb33235-bib-0064] Zimet, G. , Powell, S. , Farley, G. , Werkman, S. , & Berkoff, K. (1990). Psychometric characteristics of the multidimensional scale of perceived social support. Journal of Personality Assessment, 55, 610–617. 2280326 10.1080/00223891.1990.9674095

[brb33235-bib-0065] Zimet, G. D. , Dahlem, N. W. , Zimet, S. G. , & Farley, G. K. (1988). The multidimensional scale of perceived social support. Journal of Personality Assessment, 52(1), 30–41. 10.1207/s15327752jpa5201_2 2280326

[brb33235-bib-0066] Zimet, G. D. , Dahlem, N. W. , Zimet, S. G. , & Farley, G. K. (1988). The multidimensional scale of perceived social support. Journal of Personality Assessment, 52, 30–41. 10.1207/s15327752jpa5201_2 2280326

[brb33235-bib-0067] Zunzunegui, M.‐V. , Alvarado, B. E. , Del Ser, T. , & Otero, A. (2003). Social networks, social integration, and social engagement determine cognitive decline in community‐dwelling Spanish older adults. Journal of Gerontology: Social Sciences, 58, S93–S100. 10.1093/geronb/58.2.S93 PMC3833829 12646598

